# Public Response on Social Media to a Social Marketing Campaign for Influencing Attitudes towards Boating Safety

**DOI:** 10.3390/ijerph18126504

**Published:** 2021-06-16

**Authors:** Jennifer Smith, Tessa Clemens, Alison Macpherson, Ian Pike

**Affiliations:** 1 BC Injury Research and Prevention Unit, BC Children’s Hospital, Vancouver, BC V6H 3V4, Canada; ipike@bcchr.ca; 2 Drowning Prevention Research Centre, Toronto, ON M2J 1P8, Canada; tessakclemens@gmail.com; 3 School of Kinesiology and Health Science, York University, Toronto, ON M3J 1P3, Canada; alison3@yorku.ca; 4 Department of Pediatrics, The University of British Columbia, Vancouver, BC V6H 3V4, Canada

**Keywords:** recreational boating, drowning prevention, social marketing, Facebook

## Abstract

The purpose of this research paper is to assess the response on Facebook to a social marketing campaign for recreational boating safety. The campaign ran for the 2018 and 2019 boating seasons in British Columbia, Canada. Messages related to boating safety were delivered in multi-media formats, including ten Facebook posts. All public comments on the campaign Facebook page in response to the ads were included in the analysis. Comments were reviewed for tone and subject; those that related directly to the campaign or boating safety-related topics, such as alcohol use or enforcement, were labeled positive, negative or neutral in tone. Metrics such as likes and shares were also noted. The overall engagement rate (defined as engagements over people reached) was 4.1%. The posts were liked >7000 times and received 901 shares. A total of 219 comments were analysed. Almost half of the comments were positive (*n* = 106, 48.4%). Fifty comments were off-topic (22.8%), 45 were neutral (20.5%) and 18 were negative (8.2%). The majority of comments were positive, indicating that the campaign performed as planned and was generally well received by the people for whom it was intended. Comments illuminated prevailing attitudes towards risks, injuries and safety practices related to recreational boating. Positive comments valued safety as an aspect of having a pleasant experience, rather than a barrier. Negative comments were about perceiving reduced fun of boating, rather than objecting to the campaign itself. As a component of a multi-media social marketing strategy, Facebook can be a source of instant feedback from the campaign audience.

## 1. Background and Significance

Canada has achieved substantial declines in water-related fatality rates over the past 25 years, yet drowning remains one of the top ten causes of injury death [1,2]. From 2008 to 2017 in British Columbia (BC), an average of 75 people lost their lives to drowning each year [1]. The majority of these deaths occurred in natural bodies of water, particularly among young and middle aged adults [1]. Approximately 30% were engaged in recreational boating at the time, and 59% of these were power boats, most commonly under 5.5 m [3]. Drowning outcomes can be fatal or non-fatal. Early rescue and resuscitation improve survival, yet primary prevention remains the most impactful approach [2].

Incorrect or non-wear of lifejackets or personal floatation devices (PFDs) and alcohol consumption are well-established risk factors for recreational boating fatalities. A review of all unintentional drowning deaths in Canada from 2012 to 2016 found that, for those cases for which information about lifejackets or PFDs was available, 86% were not wearing one or wearing it incorrectly [3]. Thirty-four percent of fatalities known to not be wearing a lifejacket or PFD had one in the boat at the time of incident, but were unable to access it or put it on once in the water. Further, alcohol was a factor in 35% of recreational boating deaths [3]. The Canadian statistics reflect trends in other developed countries. A recent 10-year retrospective study (2019) of recreational boating deaths from Australia reported incorrect or non-wear of PFDs in 91% of fatalities, alcohol involvement in 29% and drug use in 30% [4]. A case-control study of recreational boating fatalities from Washington State found that those who died were 2.6 times more likely to not be wearing a lifejacket or PFD, and 70% more likely to have a fatal outcome in an incident involving alcohol, compared to those who had a non-fatal outcome [5]. Given the abundance of evidence that alcohol use and/or not wearing a lifejacket or PFD contribute to a large proportion of recreational boating deaths, both risk factors are obvious and appropriate targets for intervention. 

There are various approaches to preventing boating injuries related to alcohol use and not wearing a lifejacket or PFD, such as operator safety training, enforcement of laws and educational campaigns [6]. While there is some evidence of effectiveness, such interventions are insufficient without individual action and/or compliance, so behaviour change should be considered an essential component of any boating safety strategy [7,8,9]. One United States (US) study showed that a 20% increase in the rate of lifejacket wear would have saved 1200 lives out of 2200 drowning deaths, while a matched case control study of PFD wear found the relative risk of drowning without a PFD was one in two [10,11]. A 2018 systematic review reported that alcohol significantly increased the severity of boating injuries, with fatal outcomes more likely with alcohol use, and the risk of death increasing with increasing blood alcohol concentration (BAC) [12]. However, boaters were found to underestimate the risks to passengers of alcohol use, even with high awareness of local laws and legal limits [12]. The challenge facing prevention advocates is how to achieve consistent behaviour modification among boaters who engage in risky boating practices.

While mass media campaigns are one of the interventional options for promoting positive health behaviours, evidence of their effectiveness is mixed [13]. Social marketing goes beyond the traditional, informative approach of mass media campaigns to engage the audience and invite their active participation in shaping a new social norm for the benefit of society [14]. Because social marketing is grounded in understanding of the behavioural determinants of its intended audience, the communication strategy is uniquely tailored to a particular issue as it is experienced by that audience [15,16].

*Preventable* (www.preventable.ca, accessed on 16 June 2021) is currently the only social marketing organization in Canada focused on beliefs, attitudes and behaviours related to injury prevention. Evaluation of the campaign has demonstrated significant results in changing attitudes and behaviours among the adult population over the past ten years [17,18,19]. *Preventable* often employs social media, particularly Facebook, as part of its multi-media communications strategy. Since the launch of Facebook into the public domain in 2007, its exponential growth has dominated the social media landscape [20]. Social scientists and commercial marketers have long recognised the platform’s potential as a rich source of data that provides insights into human personality, social interactions and behaviours [21]. Health promotion practitioners have also recognised the potential of Facebook and other social media platforms for disseminating health messages, but the bidirectional interactivity that is unique to these communication channels affords researchers novel opportunities to circumvent some of the biases that affect more traditional qualitative methods and receive feedback on their interventions in real time [14,21]. With each campaign that is promoted on Facebook, *Preventable* generates a wealth of data through audience engagement on its social platform.

## 2. Objective

The purpose of this research paper is to assess the short-term public response to a targeted, evidence-based social marketing campaign using the *Preventable* brand platform and marketing approach to influence recreational boaters.

## 3. Materials and Methods

### 3.1. The Campaign

The campaign was developed in three phases and modeled on the previously-tested *Preventable* brand platform. Phase I entailed an evidence review and literature search, describing the epidemiology of recreational boating injuries in BC, reviewing the current peer reviewed literature on risk factors and interventions, as well as an overview of prevention efforts in Canada, including the current legislation with respect to lifejacket wear and alcohol consumption. The results of this comprehensive review are published elsewhere, and were used to form the basis of the marketing strategy [22].

**Figure 1:**
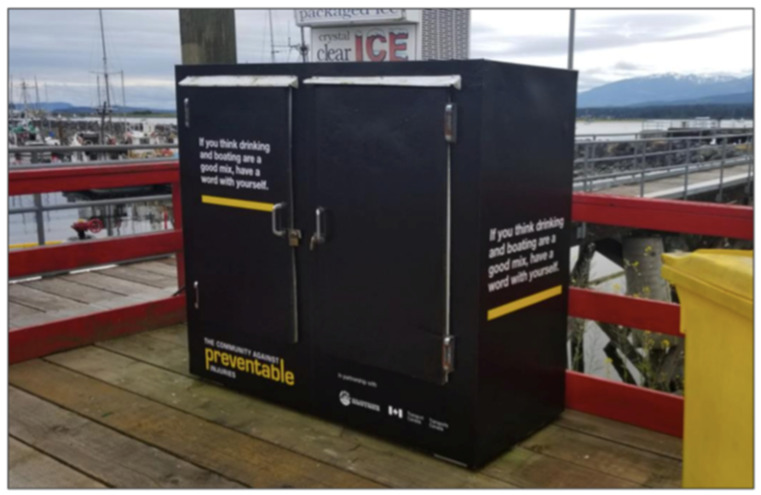
An example of campaign messaging placement at a high-traffic area in the marina.

Phases II and III were implementation and optimization phases undertaken in partnership with Royal Canadian Marine Search and Rescue (RCMSAR), a registered charity with volunteer capacity for public outreach in addition to their search and rescue mandate, and Transport Canada, the regulatory authority. The campaign creative was developed and put into market during the recreational boating season in BC (May–September) in 2018 and 2019. Messages related to drowning prevention, lifejacket wear, alcohol use and safety equipment were delivered in multi-media formats at times and in places where boaters were likely to be engaged in, or preparing for recreational boating (Figure 1). Ongoing monitoring of the campaign allowed for adjustments to the weighting of different elements at different times; for example, posts on social media were timed to be seen in the lead up to long weekends during the boating season, while marina signage remained in place throughout the campaign period in high-traffic locations. RCMSAR volunteers were briefed on the campaign and participated in dockside or on-water conversations with boaters about the campaign messaging and safety preparedness throughout the boating season.

Ten Facebook posts were published during the two campaign periods, seven in 2018 and three in 2019, as part of the multi-media campaign activities.

### 3.2. Social Media Data Analysis

All public comments that appeared on the *Preventable* Facebook page in response to the campaign ads, including memes, graphic interchange formats (GIF), emoji, text, photos, Facebook stickers or any combination of these, were included in the analysis, excluding only replies from *Preventable*. Data were abstracted from Facebook one month following the end of the campaign in September 2019. Comments were reviewed for tone and content in the context of the ad and all other comments on the post. Comments that related directly to the campaign or to the topic of boating safety were labeled positive, negative or neutral. Positive comments were those that were in some way supportive of the campaign messages and activities; for example, reiterating the need for lifejackets or relating a personal story about the dangers of alcohol use and boating. Negative comments were those that refuted the campaign message, or otherwise directly expressed or agreed with another comment expressing an unfavourable opinion of the campaign or campaign messages. Neutral comments were those that expressed neither positive nor negative sentiments in relation to the campaign, or those that could be interpreted as either. Comments that were unrelated to the campaign or topic of boating safety were counted, but not included in the analysis. Other metrics, such as likes, shares and clicks were also noted [23].

## 4. Results

Facebook metrics on each of the ten posts are shown in Table 1. Hashtags and links to partner organization websites have been removed from the post captions for brevity. The overall engagement rate (defined as engagements over people reached) for the campaign was 4.1%.

A total of 233 comments were made on the public Facebook page in response to the campaign posts in 2018 and 2019. Fourteen comments were labeled “troll”. The clear purpose of these comments was to elicit outrage, rather than to engage in genuine discussion about the topic. The comments were often personally insulting or inflammatory and were made in every case but one by the same user. Troll comments were excluded on the premise that they would be posted by the user regardless of the topic and therefore did not reflect a true position. This left 219 comments for analysis.

Almost half of the comments were positive (*n* = 106, 48.4%). Fifty comments were off-topic (22.8%), 45 comments (20.5%) were neutral and the smallest number of comments were negative (*n* = 18, 8.2%). Twenty-nine comments (27.4%) thanked the RCMSAR volunteers for their service, told a positive story about their campaign-related public engagement activities, or praised the organization. Other commenters told a personal story about boating safety in support of the campaign messages or offered safe boating tips (*n* = 9, 8.5%).

Approximately one-quarter (*n* = 12, 24.0%) of off-topic comments expressed environmental concerns, such as wildfires, pollution or protections for whales and other marine life.

One-quarter (*n* = 11, 24.4%) of neutral comments provided factual information about boating safety, clarifying regulations or correcting misconceptions in a neutral tone. Seven commenters (15.6%) expressed some variation on the sentiment that boating injuries happen because some people are inherently foolish. While this type of comment was clearly negative in tone, commenters were not expressing a negative sentiment about the campaign, prevention efforts or boating safety in general, nor were they off-topic, which is why such comments were included in the neutral category.

Some users (*n* = 7, 38.9%) had misconceptions about the role and activities of RCMSAR, which resulted in a negative opinion of the campaign, and which were often corrected by another user. Five negative comments (27.8%) were made by the same user who did not appear to believe that lifejackets are necessary or prevent drowning among strong swimmers or experienced boaters. This user seemed to feel that promoting lifejacket wear would encourage weakness and fear of the water. Other negative comments rejected perceived unnecessary limits on their freedom.

Sample comments from each category are shown in Table 2.

## 5. Discussion

Recreational boating-related injuries are preventable, and yet many effective strategies require individual boaters to modify their behaviours. Social marketing offers a set of behaviour change tools that direct program planners to understand audience perceptions and realities as a first step in designing and implementing effective campaign strategies, as well as monitoring and tracking effectiveness. As a component of the social marketing communications strategy, social media can be a source of instant feedback from the campaign audience. Social media evolves quickly, thereby challenging researchers to discover and adapt robust methods of gathering and triangulating the data to draw meaningful insights [24]. However, the influence of social media on attitudes and behaviours is undeniable, and health researchers are recognising the need to further our understanding of how social media data can improve the design and evaluation of health promotion interventions [25,26]. This research paper examined the public response on Facebook to a multi-media social marketing campaign that was designed to influence safer boating behaviours in BC. Facebook was a tool used in the campaign communication strategy, as well as a source of unsolicited feedback on the campaign messages in real time.

This study found that the campaign messages resonated with the personal experiences of many recreational boaters on Facebook, sparking conversations on social media that illuminated the nuances of prevailing attitudes towards risks, injuries and safety practices related to recreational boating. The majority of comments were positive, which indicates that the campaign performed as planned and was generally well received by the people for whom it was intended. Boaters who commented positively on the campaign appeared to value safety as an aspect of having a pleasant experience, rather than a barrier. Negative comments tended to object to reducing enjoyment of boating, rather than to the campaign itself. Commenters who objected to what they perceived as increased regulatory oversight on their activities may value freedom from restrictions as an important aspect of a fun boating experience. These objections, while expected, reflect important barriers to change that appear to be consistent with other research exploring boating safety attitudes in Canada [27,28].

In 2014, the Canadian Safe Boating Council (CSBC) undertook a quantitative survey via an online panel, followed by focus groups, to understand common attitudes and beliefs of recreational boaters across Canada [27]. Results of those studies indicated that boaters believed their knowledge of boating safety was high and perceived their risk of boating-related injuries to be low. Furthermore, barriers to lifejacket use included beliefs that a lifejacket would not make a difference in a cold-water submersion event and that boaters overall would not be likely to need a lifejacket because boating incidents are rare. Lastly, the majority of boaters expressed a belief that consuming alcohol is part of the enjoyment of boating and were not aware that drinking and boating is illegal. A follow up CSBC qualitative study with male participants in the same year explored ways to encourage lifejacket wear and abstaining from alcohol while boating. The study found that male recreational boaters valued freedom from rules, relaxation, guy bonding and personal control when they engage in boating, and these values were key motivators for consuming alcohol and not wearing PFDs.

The CSBC findings are also consistent with the peer-reviewed literature of risk perception and unsafe boating practices. A dockside survey of lifejacket use among recreational boaters in Washington State found that low lifejacket use was significantly associated with self-perceived “expert” swimming ability (RR 1.25, 95% CI 1.03 to 1.53) and possibly with the belief that a lifejacket would not save a person from drowning [28]. The same study also found that low lifejacket use was associated with alcohol consumption of any amount (RRs ranging from 1.09 to 1.13). Another survey of recreational boaters in the US found that while a minority had formal training specific to the type of boat they operated, those who were trained formally perceived their injury risk to be lower than those who were self-taught or learned from family or friends [29]. The authors were surprised to find that operator training was not associated with PFD use, and trained and untrained operators were equally likely to consume alcohol. The effects of operator training on boaters’ behaviours have not been demonstrated consistently in previous literature [29]. However, the authors hypothesized that some boating courses in the US may not adequately address the risks of alcohol and boating, and that those boaters who take a formal boating course are more likely to feel increased confidence, and therefore perceive a reduced risk to themselves while boating [29]. Together, these studies illustrate common beliefs and attitudes that present significant challenges to interventionists: when boaters believe their overall risk is low, and that wearing a lifejacket or PFD and alcohol consumption have minimal, if any, influence, why would they pay attention to campaigns that address these very behaviours?

The key to a successful and well-designed social marketing effort starts with understanding and speaking directly to the perspectives and realities of the audience segment that the campaign is intended for [30]. Audience insights have been the foundation of *Preventable’s* approach to injury prevention in British Columbia, Canada, for over ten years [18]. *Preventable’s* messaging and tone was developed after three years of consultation and research with British Columbians, and is based upon an approach they indicated would be most compelling. *Preventable* consulted widely with British Columbians to explore their knowledge, attitudes and behaviours regarding injury prevention in various risk situations, including recreational activities like boating, and learned that most people knew what to do to keep themselves safe, but tend to cut corners out of a sense of complacency. Because people do not need to be told what to do, given that they already know, a reminder in the right time and place is welcomed, particularly if it acknowledges that people are intelligent and are usually doing their best in any given situation. Further, they do not like to be shamed into changing their behaviour and indicated that this type of messaging would not hold their attention. Therefore, the boating campaign was designed to reflect these principles for the widest appeal, while challenging boaters to reflect upon their attitudes towards boating safety and take actions that align with their pre-existing knowledge.

## 6. Limitations

The intended audience for the campaign was recreational boaters in BC aged approximately 25–55 years, although boaters outside of this age range were exposed to the campaign. Social media comments that were included in the study were not screened for age and location of the commenter, and could therefore be from outside of BC or from respondents who fall outside of the target age range. However, the social media ads were targeted through Facebook’s algorithm to adult boaters in BC and it is therefore less likely that the comments included in this study reflect the views of an entirely different population [21]. Social media comments were from those who felt most compelled to respond, any may not be representative of the majority views in BC. However, they may also capture responses from those who would otherwise not be captured by other methods, such as a survey, which reflects a strength of the present study.

## 7. Conclusions

The results of the present study suggest that the *Preventable* platform is well-accepted by Facebook users as part of a multi-media boating safety social marketing strategy, and provides further support for previous Canadian research exploring attitudes that underly risky boating behaviours such as not wearing a lifejacket or PFD and alcohol use while boating. Negative comments on the campaign rejected the perceived limits on freedoms that enhance enjoyment of boating, rather than refuting the campaign messages directly. Given the availability of user-generated data on Facebook and other social media platforms, researchers in health promotion are highly interested in using this data to inform the design and evaluation of interventions, such as social marketing campaigns. This study demonstrates one method of leveraging unsolicited feedback to monitor public response to the campaign messages and assess the campaign developers’ understanding of audience insights in the setting of recreational boating in British Columbia.

## Figures and Tables

**Table 1:** Facebook metrics by campaign post.

Publish Date	Caption	People Reached ^a^	Engagements ^b^	Reactions/Comments/Shares	Likes	Comments	Shares
18-May-2018	Wondering if you have proper safety equipment on board? Royal Canadian Marine Search and Rescue volunteers are here to help. Find them out on the water this summer talking safety with BC boaters.	9319	681	489	419	6	41
18-May-2018	Boating season has finally arrived. Stay safe on the water and reduce injuries by making sure you have all the proper safety equipment on board.	6489	428	353	308	8	29
01-Jun-2018	Ready for some time on the boat? Keep everyone safe, including yourself, by wearing a lifejacket when you’re on the water.	16,060	1012	799	690	14	56
15-Jun-2018	Drowning doesn’t just “happen”—it’s almost always #preventable. Learn what you can do to protect you and your family from boating related fatalities.	13,337	882	681	555	19	90
29-Jun-2018	Royal Canadian Marine Search and Rescue volunteers will be out in BC waters this long weekend to talk to boaters about preventable injuries and staying safe on the ocean. Have you got all the right equipment?	23,054	1570	1145	976	40	76
03-Aug-2018	Be on the lookout this weekend for Royal Canadian Marine Search and Rescue volunteers as they greet boaters on the water to talk about proper boating safety equipment and staying safe this summer. Have you got all the right equipment?	48,391	2511	1532	1254	43	180
30-Aug-2018	This Labour Day long weekend, Royal Canadian Marine Search and Rescue volunteers will be on the waters speaking with BC boaters about proper boating safety equipment and staying safe on the water.	21,403	2127	1572	1311	32	157
28-Jun-2019	Drowning doesn’t just “happen”—it’s almost always #preventable. Learn what you can do to protect you and your family from boating related fatalities. Stay safe while enjoying the water this summer!	27,553	1026	860	689	37	111
02-Aug-2019	Drowning doesn’t just “happen”—it’s almost always #preventable. Learn what you can do to protect you and your family from boating related fatalities. Stay safe while enjoying the water this summer!	109,179	600	76	46	8	18
30-Aug-2019	If you think boating without lifejackets for everyone is not a big deal, have a word with yourself. PFDs are an essential part of safe boating. Stay safe	31,967	1772	1490	1189	26	143
	Totals	306,752	12,609	8997	7437	233	901

^a^ The number of users who saw the post at least once. ^b^ Includes all actions taken by users during the time that the post is live. This will include views, comments, shares, clicks or reactions.

**Table 2:** Examples of positive, neutral and negative user comments.

	Sentiment	Examples
**Positive**	Reinforcing the campaign message	“Pay attention, boaters. This is important!” “There is no such thing as accidents, only incidents. This is because everything is preventable.”
	Gratitude	“You guys do an amazing job both on the water, as well as raising awareness for boat safety. Thank you.” “Thank you for helping out those in need.”
	Safe boating tips	“Check the expiry dates on all life jackets and the fire extinguisher as well. Be sure you have water, flares and solid snack food in case of an emergency. Make certain your safety instructions and power squadron are also current. One never knows too much when it comes to boating.” “Always put life jackets on, the name speaks for itself. life.”
	Personal stories	“My cousin and his wife were with their 2 young daughters when they hit a log. Their boat was going down and he only had time to grab a girl in each arm. They had on life jackets and I know some people believe they will have time to grab their life jacket but no, you might not. Be safe not sorry.”
**Neutral**	People are foolish	“Considering all the stupid things (some) boaters do, sadly yes.” “There are a lot of stupid people (parents) on the water.”
	Clarifying or correcting	“They only rescue and inform folks. They do no enforcement of any laws.” “Pleasure Craft Operator Card—MANDATORY for operating a powered boat in BC.”
**Negative**	Misconceptions about RCMSAR role	“Can’t go boating without some good old government observation and intervention....” “Try not to give everyone huge fines and ruin their weekends”
	Lifejackets are not needed	“You don’t need a life jacket unless you don’t know how to swim. And you won’t get hypothermia unless it’s freezing outside.” “Teach them to swim… me and all my brothers and sisters and friends grew up on this ocean… nobody had life jackets nobody drowned... people are so useless today”

## Data Availability

Not applicable.
